# RIPK1-RIPK3 mediates myocardial fibrosis in type 2 diabetes mellitus by impairing autophagic flux of cardiac fibroblasts

**DOI:** 10.1038/s41419-022-04587-1

**Published:** 2022-02-14

**Authors:** Shigang Qiao, Lei Hong, Yongming Zhu, Jun Zha, An Wang, Jia Qiu, Wei Li, Chen Wang, Jianzhong An, Huiling Zhang

**Affiliations:** 1 Jiangsu Key Laboratory of Neuropsychiatric Diseases and College of Pharmaceutical Sciences; Suzhou Key Laboratory of Drugs Rearch for Prevention and Treatment of Hyperlipidemic Diseases, Department of Pharmacology and Laboratory of Cerebrovascular Pharmacology, College of Pharmaceutical Science, Soochow University, Suzhou, China; 2 Institute of Clinical Medicine Research, Affiliated Suzhou Science & Technology Town Hospital of Nanjing Medical University, Suzhou, China; 3 Department of Anesthesiology, Affiliated Suzhou Science & Technology Town Hospital of Nanjing Medical University, Suzhou, China

**Keywords:** Necroptosis, Diabetes complications

## Abstract

Receptor-interacting protein kinase 1 (RIPK1) and 3 (RIPK3) are critical regulators of programmed necrosis or necroptosis. However, the role of the RIPK1/RIPK3 signaling pathway in myocardial fibrosis and related diabetic cardiomyopathy is still unclear. We hypothesized that RIPK1/RIPK3 activation mediated myocardial fibrosis by impairing the autophagic flux. To this end, we established in vitro and in vivo models of type 2 diabetes mellitus with high glucose fat (HGF) medium and diet respectively. HGF induced myocardial fibrosis, and impaired cardiac diastolic and systolic function by activating the RIPK1/RIPK3 pathway, which increased the expression of autophagic related proteins such as LC3-II, P62 and active-cathepsin D. Inhibition of RIPK1 or RIPK3 alleviated HGF-induced death and fibrosis of cardiac fibroblasts by restoring the impaired autophagic flux. The autophagy blocker neutralized the effects of the RIPK1 inhibitor necrostatin-1 (Nec-1) and RIPK3 inhibitor GSK872 (GSK). RIPK1/RIPK3 inhibition respectively decreased the levels of RIPK3/p-RIPK3 and RIPK1/p-RIPK1. P62 forms a complex with RIPK1-RIPK3 and promotes the binding of RIPK1 and RIPK3, silencing of RIPK1 decreased the association of RIPK1 with P62 and the binding of P62 to LC3. Furthermore, inhibition of both kinases in combination with a low dose of Nec-1 and GSK in the HGF-treated fibroblasts significantly decreased cell death and fibrosis, and restored the autophagic flux. In the diabetic rat model, Nec-1 (1.65 mg/kg) treatment for 4 months markedly alleviated myocardial fibrosis, downregulated autophagic related proteins, and improved cardiac systolic and diastolic function. In conclusion, HGF induces myocardial fibrosis and cardiac dysfunction by activating the RIPK1-RIPK3 pathway and by impairing the autophagic flux, which is obviated by the pharmacological and genetic inhibition of RIPK1/RIPK3.

## Introduction

Necroptosis is a newly discovered caspase-independent mode of programed cell death that mimics the characteristics of both apoptosis and necrosis [1]. The necroptotic pathway is triggered by the receptor-interacting protein kinase 1 (RIPK1), which binds to RIPK3 via interacting with their RIP homotypic interaction motif (RHIM) domains, recruits, and subsequently phosphorylates mixed lineage kinase domain-like protein (MLKL) in the necrosome [2, 3]. Chronic hyperglycemia during type 2 diabetic mellitus (T2DM) induces cardiomyocyte death and myocardial fibrosis (MF) by accelerating proliferation of cardiac fibroblasts (CFs) and the secretion of extracellular matrix proteins. These pathological changes eventually lead to ventricular remodeling and dysfunction [4, 5]. However, the possible involvement of necroptosis and the RIPK1/RIPK3 pathway in T2DM-related MF is unclear at present.

The autophagy/lysosomal pathway is involved in cell death in response to pathological stress [6]. High glucose and fat (HGF) conditions can impair the ubiquitin–protease complex system, leading to the accumulation of damaged proteins and senescent organelles. This in turn blocks the autophagic flux and accelerates cell aging and death [7]. Consistent with this, restoring the autophagic flux clears the senescent organelles and proteins, and reduces cell death [8]. The aim of this study was to determine whether RIPK1/RIPK3 signaling is the mechanistic basis of HGF-impaired autophagic flux in diabetic myocardial fibrosis. Our findings indicate that the activation of RIPK1/RIPK3 pathway impairs the autophagic flux in CFs exposed to hyperglycemic conditions in vitro and in vivo, and inhibition of the kinases synergistically restored the autophagic flux and alleviated the HGF-induced pathological changes.

## Results

### HGF induces CFs death and fibrosis by activating the RIPK1/RIPK3 signaling pathway

**Fig. 1:**
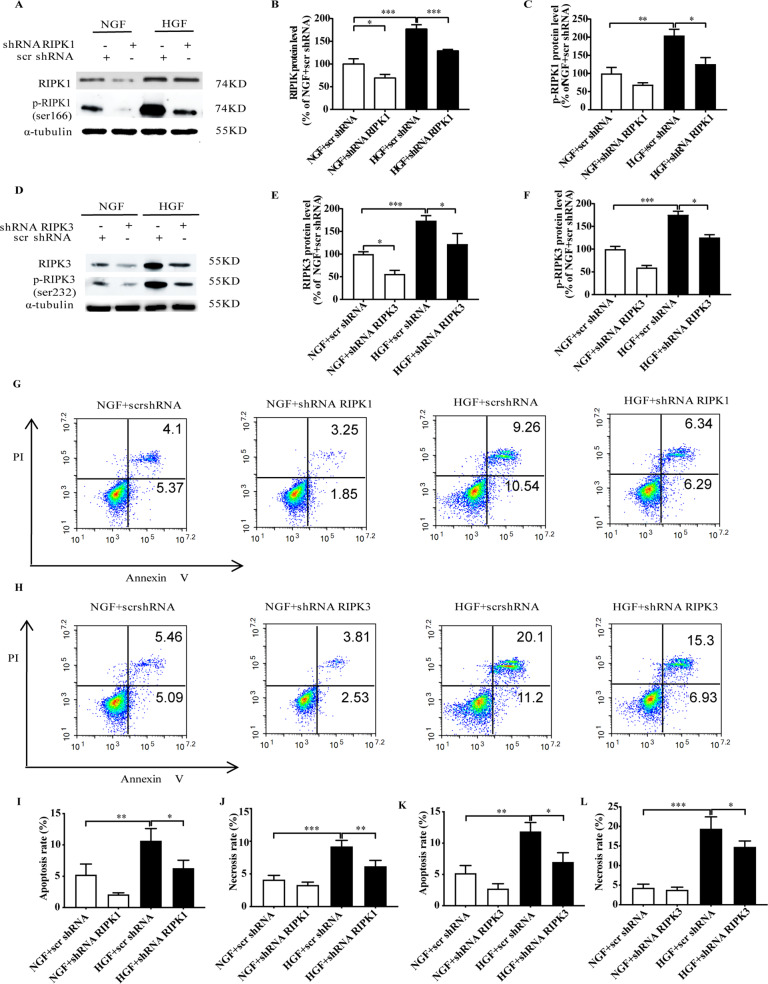
RIPK1/RIPK3 silencing inhibited HGF-induced necroptosis. Representative images and percentages of RIPK1/ser166-p-RIPK1 (**A**–**C**) and RIPK3/ser232-p-RIPK3 (**D**–**F**). Percentages of apoptotic and necrotic cells (**G**–**L**). *n* = 3 per group. Means ± SD. *P < 0.05, **P < 0.01, ***P < 0.001. NGF normal glucose and fat group, HGF high glucose and high fat group.

HGF significantly up-regulated RIPK1, p-RIPK1 (Fig. 1A–C), RIPK3, and p-RIPK3 (Fig. 1D–F) in the CFs in vitro. HGF conditions also increased CFs death and fibrosis, as indicated by excessive LDH leakage, increased levels of collagen I, collagen III, and α-SMA, and decreased ATP content (Supplementary Fig. 1). Furthermore, HGF also increased apoptosis and necrosis rates of the CFs (Fig. 1G–L). RIPK1/RIPK3 signaling was inhibited with shRNAs targeting RIPK1 (Fig. 1A–C) or RIPK3 (Fig. 1D–F). Silencing of either RIPK1 or RIPK3 decreased the apoptosis and necrosis rates (Fig. 1G–L), LDH leakage, the levels of collagen I, collagen III, α-SMA, and increased the ATP content in the HGF-treated CFs (Supplementary Fig. 1). In addition, Silencing of MLKL decreased LDH leakage, the levels of collagen I, collagen III, and increased the ATP content (Supplementary Fig. 2). Taken together, RIPK1/RIPK3 inhibition can abrogate the pathological effects of HGF.

### RIPK1/RIPK3 inhibition restores the autophagic flux in the HGF-treated CFs

**Fig. 2:**
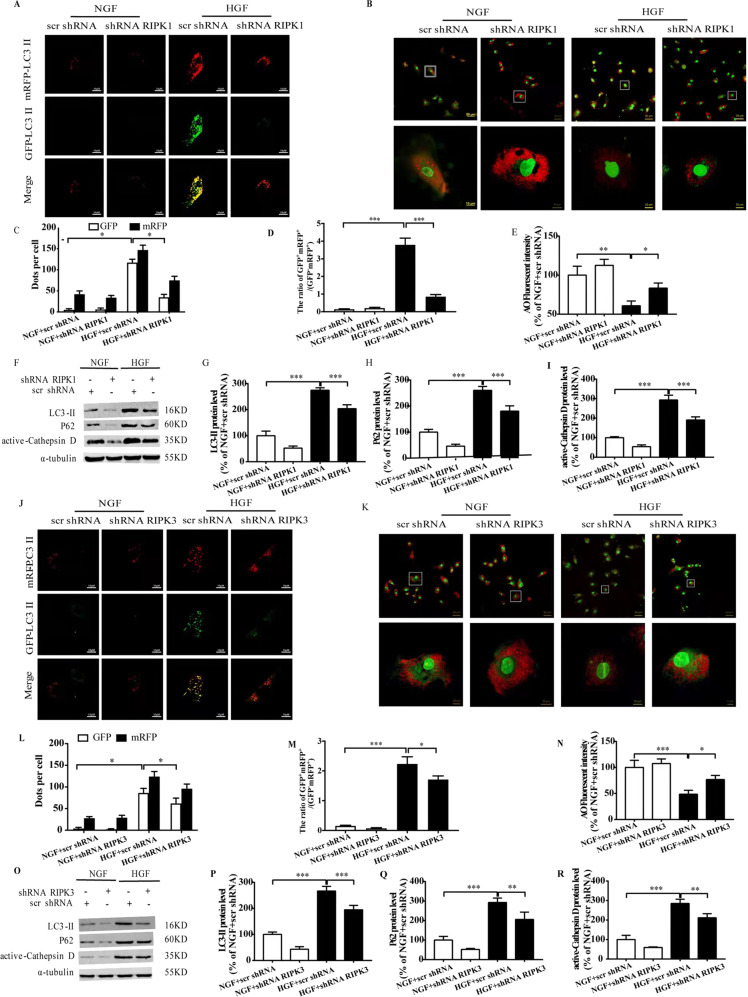
RIPK1/RIPK3 silencing restored autophagic flux in HGF-treated CFs. Confocal images and the ratio of GFP^+^mRFP^+^/(GFP^-^mRFP^+^) and AO fluorescence intensity (**A**–**E**, **J**–**N**); Representative images and percentages of LC3-II, P62, active-cathepsin D (**F**–**I**, **O**–**R**). *n* = 3 per group. Means ± SD. *P < 0.05, **P < 0.01, ***P < 0.001. NGF normal glucose and fat group, HGF high glucose, and high fat group, AO acridine orange.

**Fig. 3:**
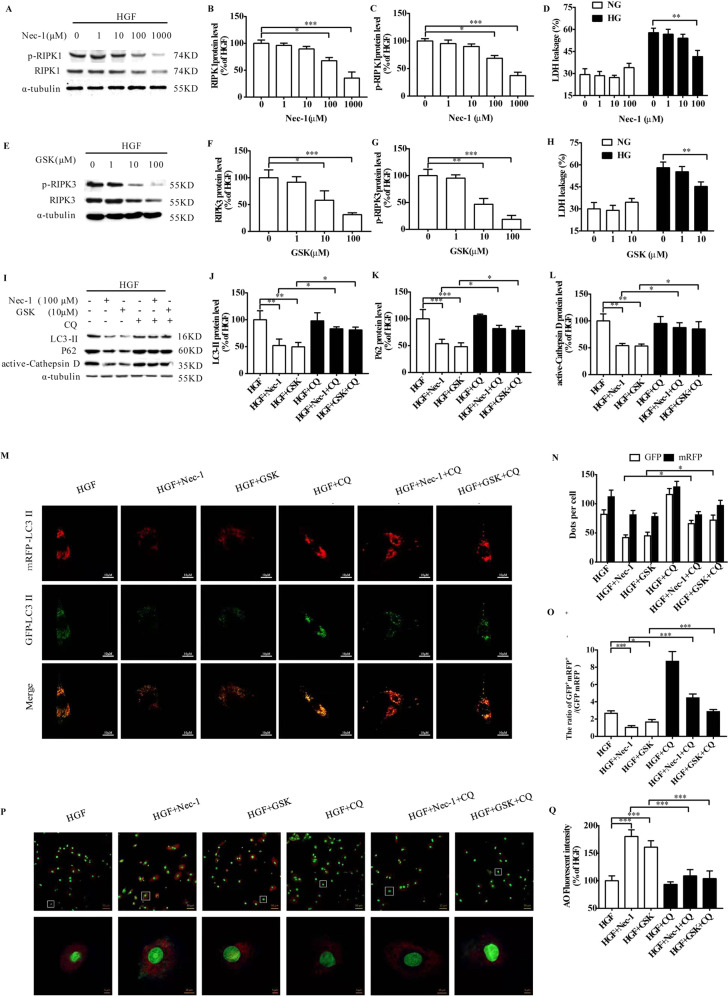
Pharmacological blockade of autophagy neutralizes the effects of RIPK1/RIPK3 inhibition. Representative images and percentages of RIPK1/ser166-p-RIPK1 (**A**–**C**), RIPK3/ser232-p-RIPK3 (**E**–**G**), and LDH leakage (**D**, **H**) following treatment Nec-1 (100 μM) and GSK (10 μM). Representative images and percentages of LC3-II, P62, active-cathepsin D (**I**–**L**); Confocal images and the ratio of GFP^+^mRFP^+^/(GFP^-^mRFP^+^) and AO fluorescence intensity (**M**–**Q**). *n* = 3 per group. Means ± SD. *P < 0.05, **P < 0.01, ***P < 0.001. NGF normal glucose and fat group, HGF high glucose, and high fat group, Nec-1 necrostatin-1, GSK GSK872, CQ, chloroquine, AO acridine orange.

HGF impaired the autophagic flux in CFs, as indicated by raised GFP^+^mRFP^+^/(GFP^-^mRFP^+^) ratio and lowered AO fluorescence intensity (Fig. 2A–E), as well as increased expression of LC3-II, P62, and active-cathepsin D (Fig. 2F–I). RIPK1/RIPK3 silencing restored the autophagic flux in the HGF-treated CFs in terms of the aforementioned indices (Fig. 2). Nec-1 inhibits the phosphorylation of RIPK1 by blocking its active fragment, and GSK has a similar inhibitory effect on RIPK3 [9]. As showed in Fig. 3, 100 μM and 1000 μM Nec-1 significantly inhibited RIPK1 and p-RIPK1 in the HGF-treated cells, and prevented LDH leakage (Fig. 3A–D). Similarly, 10 μM and 100 μM GSK alleviated LDH leakage by inhibiting RIPK3 and p-RIPK3 (Fig. 3E–H). However, since 1000 μM Nec-1 and 100 μM GSK significantly increased cell death in the absence of HGF (data not shown), we used 100 μM Nec-1 and 10 μM GSK for the subsequent experiments. At these doses, each treatment prevented HGF-induced cell death and fibrosis (Supplementary Fig. 3A–F), restored the autophagic flux, and decreased autophagic related proteins expression (Fig. 3I–Q), and their effects were neutralized by the autophagy blocker chloroquine (CQ).

### Low dose of Nec-1 and GSK alleviates HGF-induced CF death by restoring the autophagic flux

**Fig. 4:**
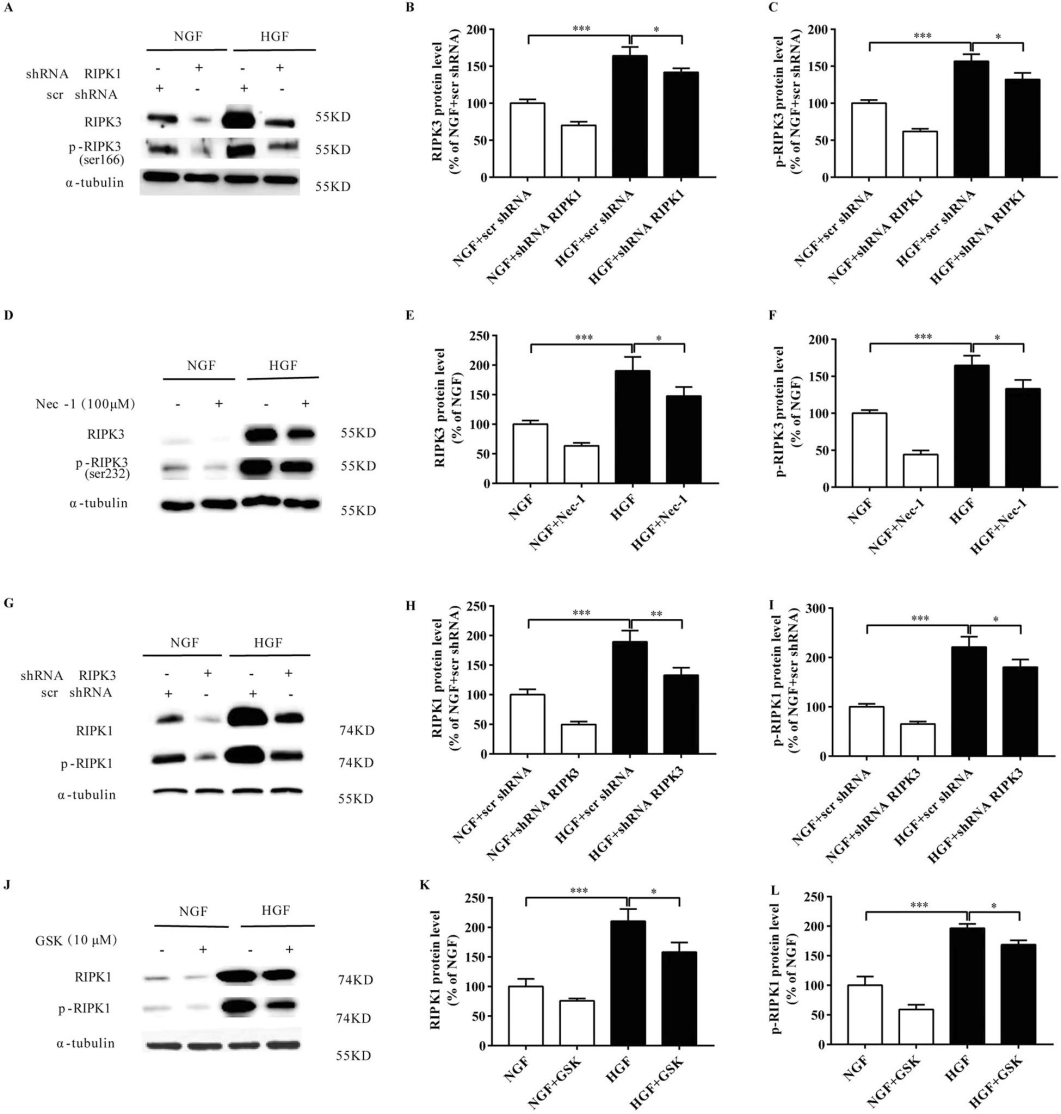
RIPK1/RIPK3 inhibition represses respectively RIPK3/ser232-p-RIPK3 or RIPK1/ser166-p-RIPK1. Representative images and percentages of RIPK3 and ser232-p-RIPK3 (**A**-**F**); Representative images and percentages of RIPK1 and ser166-p-RIPK1 (**G**-**L**). *n* = 3 per group. Means ± SD. *P < 0.05, **P < 0.01, ***P < 0.001. NGF normal glucose and fat group, HGF high glucose and high fat group, Nec-1 necrostatin−1, GSK GSK872.

**Fig. 5:**
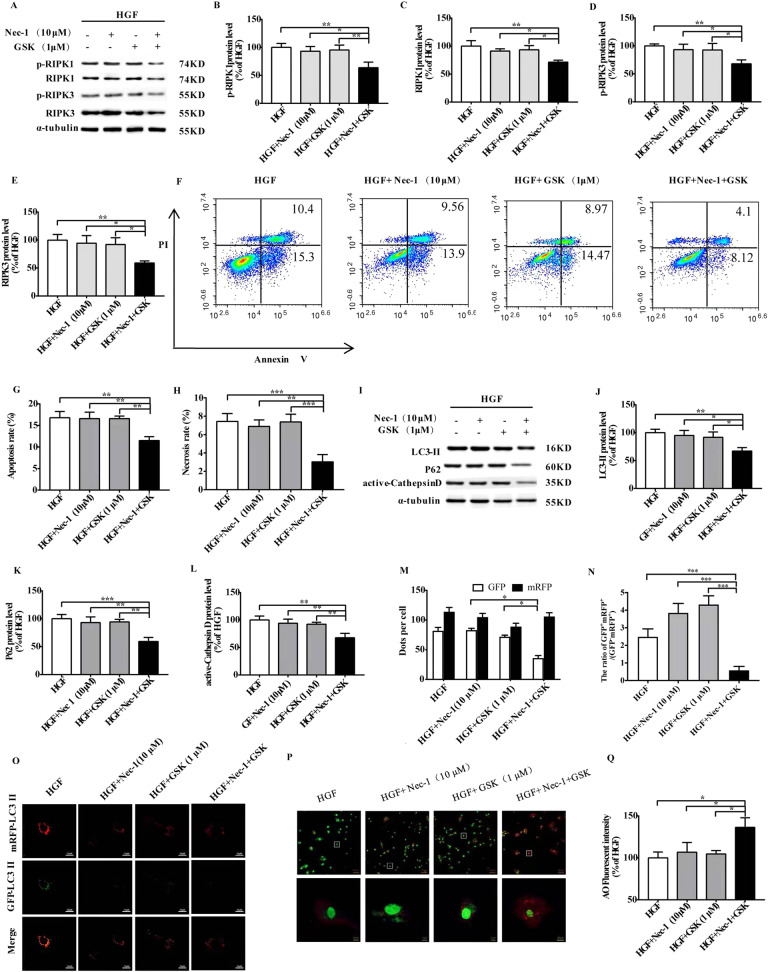
The combination of low-dose RIPK1 and RIPK3 inhibitors alleviates cell death and restores autophagic flux. Combination of Nec-1 (10 μM) and GSK (1 μM) down-regulates the levels of RIPK1, ser166-p-RIPK1, RIPK3, ser232-p-RIPK3, alleviates cell death and restores autophagic flux of CFs. Representative images and percentages of RIPK1, ser166-p-RIPK1, RIPK3, ser232-p-RIPK3 (**A**–**E**); flow cytometry analysis results (**F**–**H**); Confocal images and the ratio of GFP^+^mRFP^+^/(GFP^-^mRFP^+^) and AO fluorescence intensity (**I**–**M**); Representative images and percentages of LC3-II, P62, active-cathepsin D (**N**–**Q**); *n* = 3 per group. Means ± SD. *P < 0.05, **P < 0.01, ***P < 0.001. NGF normal glucose and fat group, HGF high glucose and high fat group, Nec-1 necrostatin-1, GSK GSK872, AO acridine orange.

RIPK1/p-RIPK1 (Fig. 4A–F) and RIPK3/p-RIPK3 (Fig. 4G–L) expression levels were markedly suppressed with the specific shRNAs and small molecule inhibitors (Nec-1 and GSK, respectively). The combination of low dose Nec-1 (10 μM) and GSK (1 μM) significantly down-regulated the levels of RIPK1, p-RIPK1, RIPK3 and p-RIPK3 (Fig. 5A–E), decreased LDH leakage and the levels of collagen I, collagen III and α-SMA, increased ATP content (Supplementary Fig. 3G–L), and decreased the number of apoptotic and necrotic cells (Fig. 5F–H). Furthermore, the combination of Nec-1 and GSK decreased HGF-induced the up-regulation of LC3-II, P62, active-cathepsin D (Fig. 5I–L), as well as lowered the GFP^+^mRFP^+^/(GFP^-^mRFP^+^) ratio and increased in AO fluorescence intensity (Fig. 5M–Q). In addition, whether the silencing of RIPK1 affects the association of RIPK1 with P62 and the binding of P62 to LC3 were examined, the results of immunoprecipitates showed that both the association of RIPK1 with P62 and the binding of P62 to LC3 were increased in HGF treated CFs. The silencing of RIPK1 significantly decreased the association of RIPK1 with P62 and the binding of P62 to LC3 (Supplementary Fig. 4). Collectively, simultaneous inhibition of RIPK1 and RIPK3 can restore the autophagic flux impaired by HGF.

### Nec-1 alleviates MF in diabetic rats

**Fig. 6:**
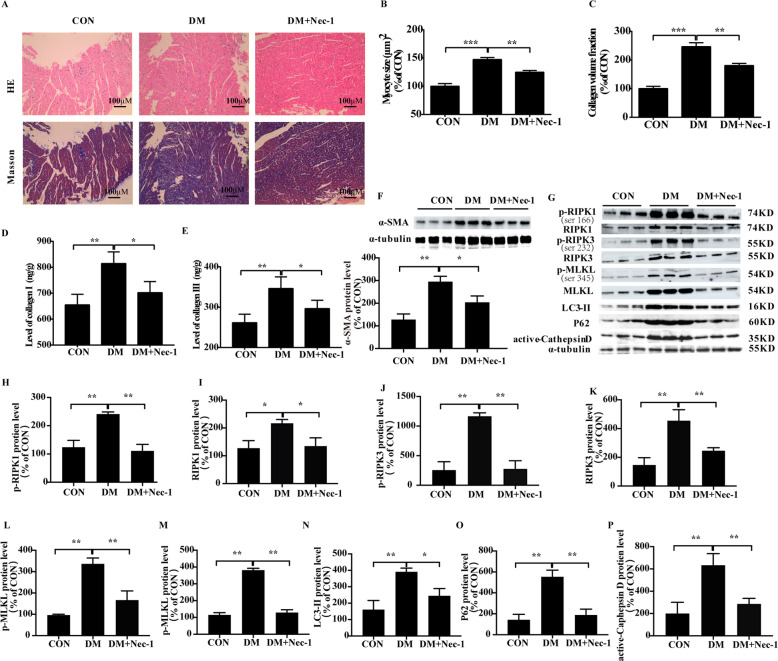
Nec-1 alleviates cardiac dysfunction in diabetic rats. Myocyte size and collagen volume fraction (**A**–**C**). The levels of collagen I and collagen III (**D**, **E**). Representative images and percentages of α-SMA, RIPK1, RIPK3, MLKL, ser166-p-RIPK1, ser232-p-RIPK3, ser345-p-MLKL, LC3-II, P62 and active-cathepsin D (**F**–**P**); Scale bar = 100 μm. *n* = 3 per group. Means ± SD. *P < 0.05, **P < 0.01, ***P < 0.001. DM group. CON control group, DM diabetic mellitus group, HE hematoxylin-eosin staining, α-SMA α smooth muscle actin.

Diabetes was induced in a rat model with a combination of HGF diet and STZ. Compared to the age-matched controls, 24-week-old diabetic animals showed increased plasma glucose levels and a decrease in body weight. The insulin sensitivity index was also higher in the diabetic model, although the level of fasting insulin was not significantly increased. Furthermore, HGF diet resulted in significant cardiac dysfunction characterized by lower heart weight but increased heart/body weight ratio (Supplementary Fig. 5), along with lower LV end diastolic volume and systolic volume, and longer isovolumic relaxation time (Table 1). However, Nec-1 treatment markedly decreased the heart/body weight ratio and insulin sensitivity index in the diabetic rats, and restored the aberrant myocardial parameters. In addition, Nec-1 also significantly decreased the myocyte size, collagen volume fraction, and the deposition of collagen I, collagen III and α-SMA in the cardiac tissues of diabetic rats (Fig. 6A–F). Mechanistically, Nec-1 significantly down-regulated RIPK1, p-RIPK1, RIPK3, p-RIPK3, MLKL, p-MLKL, LC3-II, P62, and active-cathepsin D as observed in the in vitro experiments (Fig. 6G–P). In summary, Nec-1-dependent inhibition of RIPK1/RIPK3 is a viable treatment strategy for diabetic cardiomyopathy.

**Table 1:** Nec-1 improves cardiac function parameters in T2DM rats.

Echocardiographic parameters	CON	DM	DM + Nec-1
Heart rate, bpm	354 ± 14	357 ± 16	362 ± 13
Anterior wall at end diastole, mm	8.37 ± 0.11	7.58 ± 0.25*	7.93 ± 0.17
Anterior wall at end systole, mm	5.59 ± 0.06	4.89 ± 0.14	5.32 ± 0.17
Posterior wall at end diastole, mm	1.80 ± 0.13	1.64 ± 0.22	1.66 ± 0.26
Posterior wall at end systole, mm	1.94 ± 0.15	1.73 ± 0.16	1.76 ± 0.12
LV end-diastolic volume, µl	458.32 ± 16.45	376.64 ± 13.63*	423.76 ± 12.73^#^
LV end-systolic volume, µl	166.68 ± 12.84	136.64 ± 10.82*	154.83 ± 11.25^#^
Ejection fraction, %	72 ± 6	67 ± 6	68 ± 5
Peak E, cm/s	1578 ± 38	1478 ± 52	1547 ± 41^#^
Peak A, cm/s	1509 ± 25	1583 ± 36	1552 ± 31
Mitral E/A ratio	1.75 ± 0.23	1.33 ± 0.23	1.38 ± 0.27
Ejection time,ms	47.57 ± 2.64	51.61 ± 2.72	48.93 ± 2.45
Isovolumic relaxation time, ms	36.82 ± 1.85	45.74 ± 2.97*	38.23 ± 2.45^#^

Means ± SD, n = 10. ^*^P < 0.05 vs. CON. ^#^*P* < 0.05 vs. DM.

## Discussion

We found that HGF induced myocardial fibrosis and cardiac dysfunction, which corresponded to RIPK1/RIPK3 activation and up-regulation of autophagic related proteins in both in vitro and in vivo models. RIPK1/RIPK3 inhibition decreased HGF-induced the up-regulation of autophagic related proteins LC3-II, P62, active-cathepsin D. In addition, inhibition of either kinase prevented HGF-induced CFs death and fibrosis, restored the autophagic flux, and the pharmacological inhibition of both kinases had a synergistic effect. However, the autophagy blocker CQ neutralized the effects of RIPK1/RIPK3 inhibition. Our findings suggest that the RIPK1/PIP3K pathway mediates cardiotoxic effects of HGF by impairing the autophagic flux, and is a promising therapeutic target in diabetes mellitus induced MF.

Pathological conditions can induce cell death via necrosis, apoptosis, autophagy etc. [10]. Necroptosis is a type of programmed cell death that is activated in the absence of intracellular apoptotic signaling [1], and is controlled by RIPK1 and RIPK3 [2]. RIPK1 forms a complex with TNF receptor-associated death domain protein, Fas-associated death domain and caspase-8, and induces necroptosis upon binding to RIPK3 by co-phosphorylation through homologous domains [11]. Kang et al. showed that activation of aldehyde dehydrogenase 2 prevented fibrosis, apoptosis, and necroptosis in primary cardiomyocytes cultured under high glucose conditions, which corresponded to a significant decrease in the expression levels of RIPK1, RIPK3, and MLKL [12]. In our study, RIPK1, RIPK3, and their phosphorylated forms were up-regulated in HGF-treated CFs and the myocardial tissues of diabetic rats, and inhibition of this pathway abrogated the pathological effects of HGF, suggesting the involvement of necroptosis in diabetes mellitus induced MF.

Autophagy is crucial to cellular homeostasis and survival [13, 14]. A number of studies have reported a possible link between autophagy and necroptosis. For instance, necrosome assembly and necroptosis induced by autophagosome accumulation in hypoxic cardiomyocytes and myocardium [15]. RIPK1 is the first discovered molecule in the necroptosis pathway [16], it contributes to neuronal and astrocytic cell death in ischemic stroke via activating autophagic-lysosomal pathway [17]. Functional suppression of RIPK1 blocks the NF-κB signaling pathway and induces neuron autophagy after traumatic brain injury [18]. On the other hand, autophagy-initiating kinase the Ser/Thr kinase unc-51 like kinase 1 controls RIPK1-mediated cell death [19]. Suppressed autophagic flux contributes to cardiomyocyte death by activating necroptotic pathways [20]. The diabetic myocardium shows high expression of autolysosomal proteins such as LC3-II, P62 and active-cathepsin D, which is indicative of an increased number of autophagosomes and blocked autophagic flux [21, 22]. The present results of mRFP-GFP-LC3 fluorescence probe and AO staining indicated that HGF impaired the autophagic flux in CFs, which was also supported by the increased expression of LC3-II, P62, and active-cathepsin D. These results provide novel insights for the diabetes mellitus induced MF.

Previous studies have shown that 7-Cl-Nec-1 down-regulated LC3-II in the ischemic brain [1], the inhibition of RIPK1 by either Nec-1 or analog construct lysosomal membrane permeability and suppressing necroptosis via autophagic pathway [17, 23]. Li et al. showed that P62 forms a complex with RIPK1-RIPK3 (necrosome) and promotes the binding of RIPK1 and RIPK3, while blocking autophagic flux promoted hypoxia/reoxygenation-evoked cardiomyocyte necroptosis [24]. Furthermore, P62-RIPK1-RIPK3-dependent necroptosis contributes to aging-related myocardial vulnerability to I/R injury and sorafenib induces DU145 prostate cancer cells death by exploring the association of P62 and RIPK1 via immunoprecipitation or a proximity ligation assay [24, 25]. Thus, the aforementioned researches indicate that sequestration of P62 from its interaction with LC3-II by P62-RIPK1 interaction possibly underlies the suppressed autophagy. The current results also demonstrated that HGF treatment markedly enhanced a physical association between P62 and RIPK1 and the binding of P62 to LC3, and these associations were inhibited by silencing of RIPK1. Furthermore, the inhibition of RIPK1 and/or RIPK3 restored the autophagic flux in the HGF-treated CFs, whereas blocking autophagy with CQ neutralized the beneficial effects of RIPK1/RIPK3 inhibition. Thus, the RIPK1/RIPK3 pathway is a promising target for alleviating MF induced by hyperglycemia.

RIPK1–RIPK3 binding in the necroptotic complex depends on the phosphorylation of RHIM domain. The RIPK1-RIPK3 complex then binds to and phosphorylates MLKL, and triggers necroptosis [26, 27]. We found that RIPK1 inhibition through gene silencing as well as pharmacological blockade also down-regulated RIPK3 and p-RIPK3, which can be attributed to the decreased expression levels of total RIPK1 and the subsequent decrease in binding to RIPK3 via the RHIM domain [26]. Likewise, inhibition of RIPK3 also repressed the levels of RIPK1 and p-RIPK1. Previous studies showed that Nec-1 alleviated sepsis-induced liver damage [28] and mitigated paraquat-induced cardiac dysfunction by inhibiting the RIPK1-RIPK3 interaction [29]. Furthermore, GSK reversed LPS-induced NLRP3 activation and lung injury by inhibiting RIPK1/RIPK3 and RIPK1-NLRP3 interaction in THP-1 cells [30]. Given the similar mechanism and different regulation of RIPK1 and RIPK3 inhibitors, the simultaneous inhibition of both kinases may have synergistic effects. Indeed, combination of low dose of Nec-1 and GSK significantly reduced cell death and fibrosis index of CFs treated with HGF, and restored the autophagic flux, whereas either inhibitor had minimal effects on these indices. Furthermore, Nec-1 significantly improved cardiac function in the HGF diet-fed diabetic rat model by restoring the autophagic flux in the myocardium. Zhu et al. [31] similarly found that Nec-1 improved renal function and inhibited necroptosis in a rat model of diabetic nephropathy. Thus, the combination of low-dose RIPK1 and RIPK3 inhibitors is an effective strategy against diabetes mellitus induced MF.

## Conclusion

Genetic silencing and pharmacological inhibition of RIPK1/RIPK3 signaling alleviate HGF-induced CFs death and fibrosis in the in vitro and in vivo models by restoring autophagic flux. Although inhibition of the RIPK1-RIPK3 necrosome with the combination of low dose of RIPK1 and RIPK3 inhibitors is an effective strategy against diabetes mellitus induced MF, there are several limitations in this study that ought to be addressed. For instance, we only tested Nec-1 in the in vivo model, and the optimal dosage of GSK needs to be determined. Secondly, the key targets of RIPK1 and RIPK3 that regulate the autophagic flux need to be further studied.

## Methods and methods

### CFs culture

Three-day-old neonatal rats were anesthetized with 4.5% isoflurane, and their hearts were quickly removed under sterile conditions. The ventricular tissues were digested and filtered. The CFs were isolated and cultured with normal glucose/fat (NGF, 5.5 mM glucose) or high glucose fat (HGF, 25 mM glucose + 200 nM palmitic acid) medium for 48 h [32, 33]. In vitro, RIPK1 inhibitor necrostatin-1 (Nec-1, Selleck, USA) or RIPK3 inhibitor GSK 872 (GSK, Glpbio, USA) was dissolved in 0.1% DMSO, CFs was incubated with Nec-1 or GSK for 48 h.

### Establishment of the T2DM rat model

The Sprague-Dawley male rats were randomly divided into the control (CON, *n* = 10) and diabetic (DM, *n* = 10) groups and respectively fed the normal chow and HGF diet (60% fat, 20% protein, 20% carbohydrates; Greisway Biotechnology Co. Ltd., Suzhou, China) for 4 weeks. The animals in the DM group were then injected intraperitoneally with streptozotocin (STZ 50 mg/kg, Sigma, USA), and citric acid buffer was given to the CON group. Fasting blood glucose (FBG) levels were measured after STZ injection, and the diabetic animals with FBG > 16.7 mmol/L were fed the HGF diet for another 16 weeks [34, 35] with or without daily intraperitoneal injections of 1.65 mg/kg Nec-1 (100 mg dissolved in 606 μl 0.01% DMSO and diluted with 4% β-cyclodextrin) [31]. The animals were euthanized 16 weeks later with an intraperitoneal injection of pentobarbital sodium. The fasting insulin level was measured using a specific kit as per the instructions, and the insulin sensitivity index was calculated as ln (fasting blood glucose × feed insulin)^−1^. The heart tissues were also weighed and the organ weight relative to the body weight was calculated. All treatments and subsequent analyses were blinded for intervention.

### Lentiviral transduction

Lentiviral vectors expressing RIPK1, RIPK3 and the respective scrambled controls were purchased from Genechem Co. Ltd. (Shanghai, China). The shRNA sequences are as follows: shRNA RIPK1: 5′-GCACAACCAGTCATGGAAA-3′

scrRIPK1: 5′-TTCTCCGAACGTGTCACGT-3′

sh RNA RIPK3: 5′-ACGGAAAGGCTTCTAAAGCAAGTGATGTT-3′

scrRIPK3: 5′-GGGTGAACTCACGTCAGAA-3′

### Echocardiography

The animals were anesthetized with 1–2% isoflurane at the end of the experimental period and echocardiography was performed as previously described [36]. The averages of three consecutive cycles were calculated.

### Histological staining

Rat hearts were isolated and fixed, embedded in paraffin, and cut into 5 μm thick sections. Masson staining was performed as previously described to evaluate the degree of myocardial injury and fibrosis [37].

### mRFP-GFP-LC3 transfection

The autophagic flux was detected using the adenovirus probe mRFP-GFP-LC3 [38]. The transfected cells were fixed with 4% paraformaldehyde. Autophagic flux was observed under Zeiss microscope (Carl Zeiss, Germany), and calculated on the basis of the fluorescent green and red puncta.

### Acridine orange (AO) staining

Lysosomal membrane permeability was evaluated using the AO (Sigma Aldrich, USA) fluorophore [39], which emits red fluorescence in the acidic secondary lysosomes and diffuse green fluorescence in the cytoplasm [40]. The suitably treated cells were incubated 5 μg/ml AO for 15 min, and observed under a confocal laser scanning microscope (Carl Zeiss, Germany).

### Western blotting

Proteins extracted from the cells/tissues were separated by SDS-PAGE and transferred to PVDF membranes. The latter were incubated overnight with primary antibodies against α-SMA (1:1000, ab7817, Abcam, UK), RIPK1 (1:1000, SAB3500420, Sigma, USA), p-RIPK1 (Ser166, 65746, 1:1000, Cell Signaling, USA), RIPK3 (1:1000, ab56164, Abcam, UK), p-RIPK3 (Ser232, 1:2000, AF7443, Affinity Biosciences, USA), MLKL (1:1000, ab194699, Abcam, UK), p-MLKL (Ser345, 1:2000, ab196436, Abcam, UK), LC3 (1:1000, L7543, Sigma, USA), P62 (1:1000, 18420-1-AP, Proteintech, China), active-Cathepsin D (1:1000, ab826, Abcam, UK) and α-tubulin (1:1000, ab7291, Abcam, UK) at 4 °C. Immunoreactive bands were visualized by enhanced chemiluminescence using the Amersham Imager 680 (GE, USA), and their densities were measured with Image J software (NIH, USA).

### Immunoprecipitation

Immunoprecipitation was performed as previously described [23].

### Flow cytometry

The suitably treated cells were harvested and washed twice with chilled PBS. The cells were resuspended in 100 μl 1× binding buffer, and stained with annexin FITC and PIat 4 °C for 30 min in the dark. The stained cells were diluted with 900 μl 1× binding buffer and acquired by flow cytometry [41].

### ELISA

Myocardial tissue samples or CFs were harvested, frozen, and homogenized in lysis buffer. The levels of fasting insulin, collagen I and collagen III (Yuanye Biotechnology Co. Ltd., Shanghai, China), lactate dehydrogenase (Beyotime Biotechnology Co. Ltd., Shanghai, China), and ATP (Jiancheng Bioengineering Institute, Nanjing, China) in the homogenates were measured using specific kits. All absorbance values were within the linear range of the standard curve and standardized with the total protein concentration.

### Statistical analysis

GraphPad Prism 7.0 (GraphPad Software, Inc, San Diego, USA) was used for statistical processing. All data are expressed as mean ± SD, and minimum three biological replicates were used. One-way analysis of variance (ANOVA) and Bonferroni post hoc test were used to compared protein expression levels, apoptosis, and necrosis rates, GFP-LC3/mRFP-LC3 ratios, fluorescence intensity of AO, myocyte size and collagen volume fraction, OD values, and various biochemical and histological indices. Two-way repeated-measures ANOVA and Bonferroni post hoc test were used to compare body weight, blood glucose, heart weight, heart/body weight, and echocardiographic data. *P* < 0.05 was considered statistically significant.

## Supplementary information

Supplementary figure 1

Supplementary figure 2

Supplementary figure 3

Supplementary figure 4

Supplementary figure 5

Supplementary figure Legend

## Data Availability

The datasets used and/or analyzed during the current study are available from the corresponding author on reasonable request.
