# Supplementary figures and images for: RIPK1-RIPK3 mediates myocardial fibrosis in type 2 diabetes mellitus by impairing autophagic flux of cardiac fibroblasts
Source: Cell Death Dis. 2022 Feb 14;13(2):147. doi: 10.1038/s41419-022-04587-1 (PMC8844355; doi:10.1038/s41419-022-04587-1)

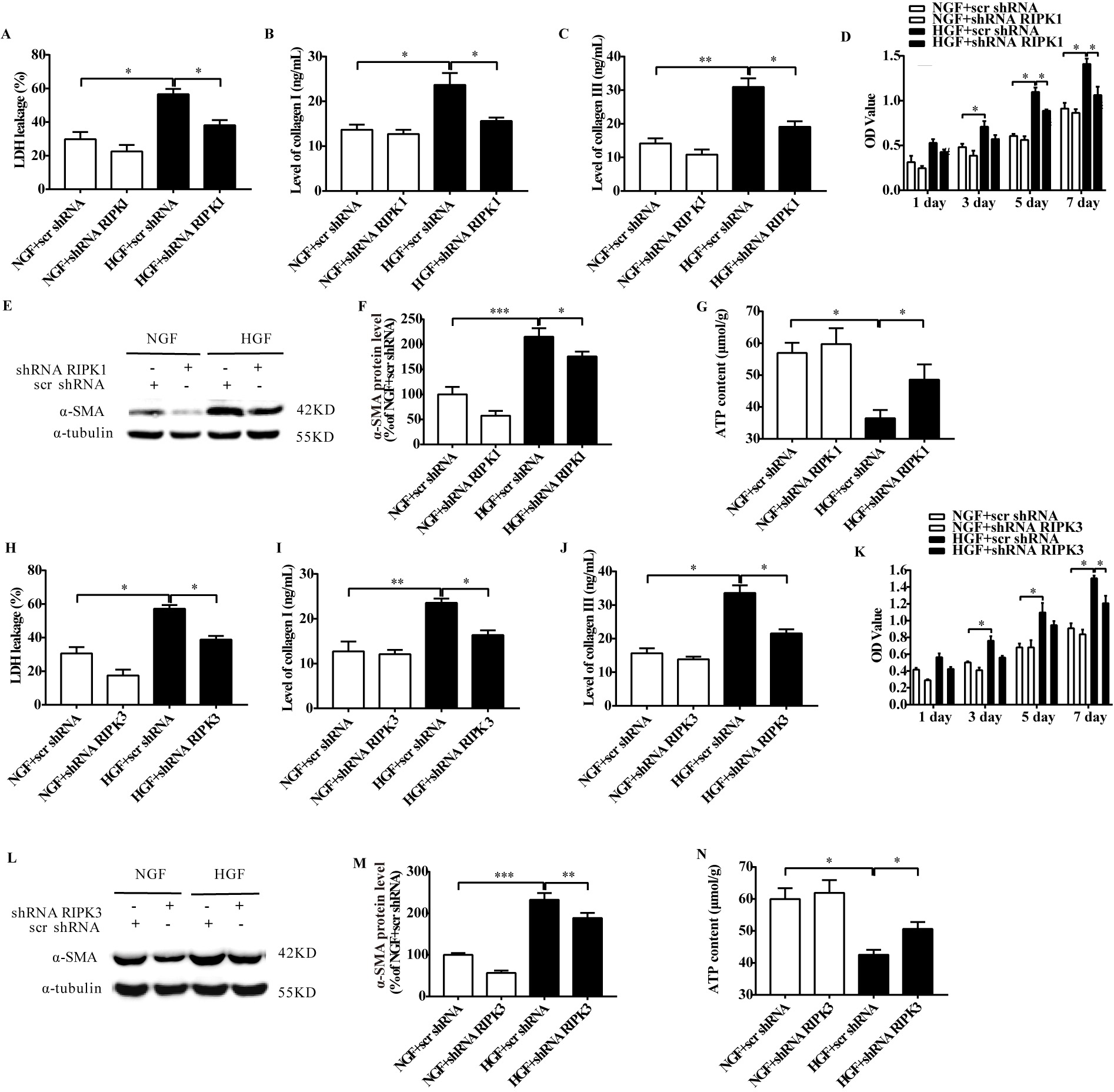

Supplement: Supplementary file 1 — Supplementary figure 1 [file 41419_2022_4587_MOESM1_ESM.jpg]

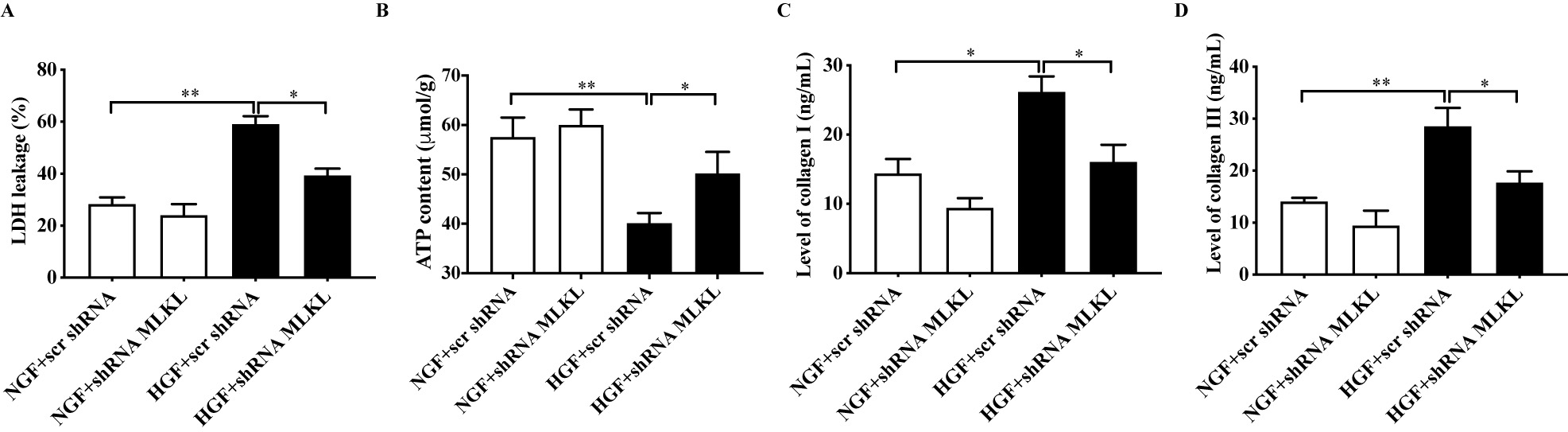

Supplement: Supplementary file 2 — Supplementary figure 2 [file 41419_2022_4587_MOESM2_ESM.jpg]

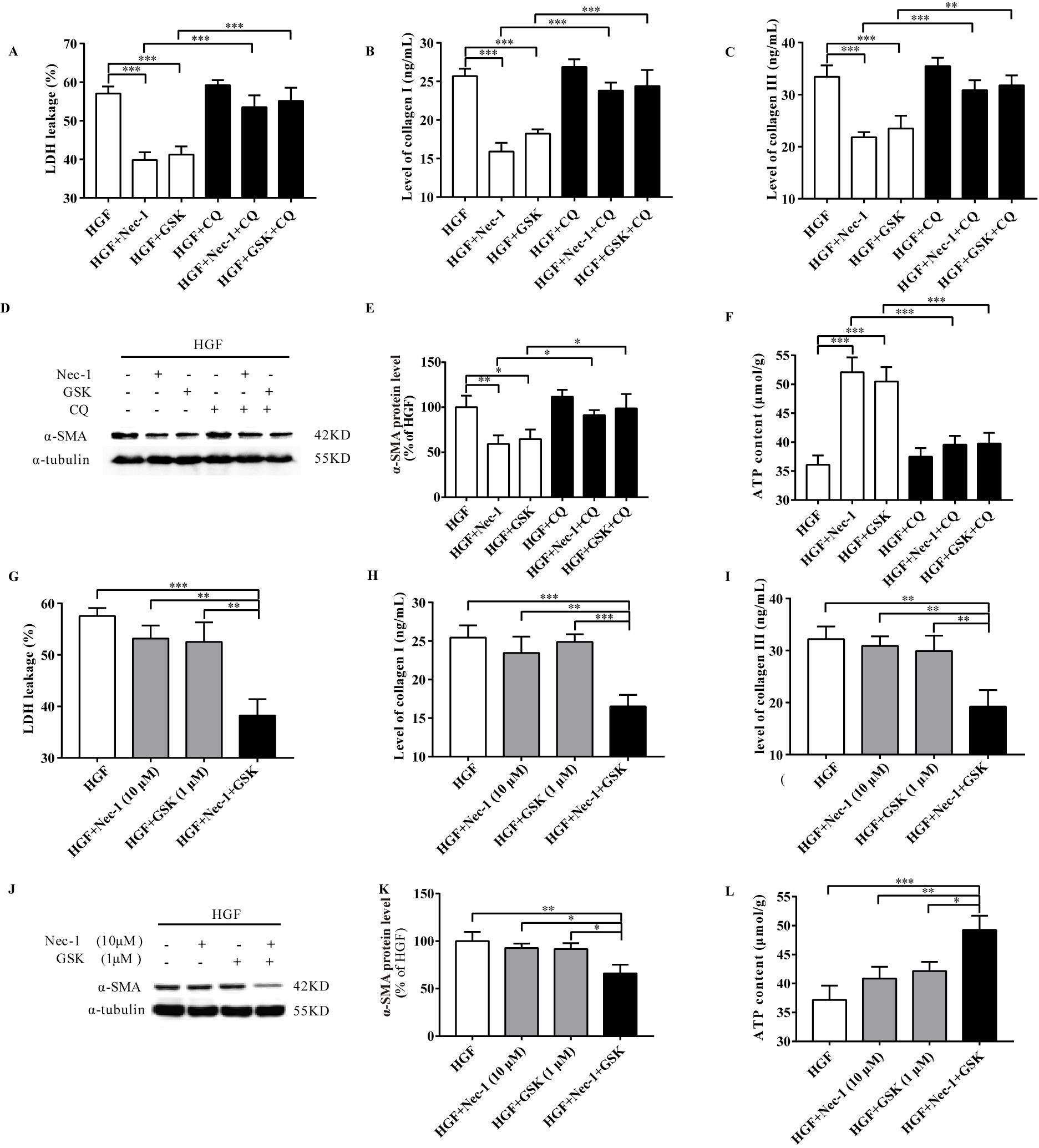

Supplement: Supplementary file 3 — Supplementary figure 3 [file 41419_2022_4587_MOESM3_ESM.jpg]

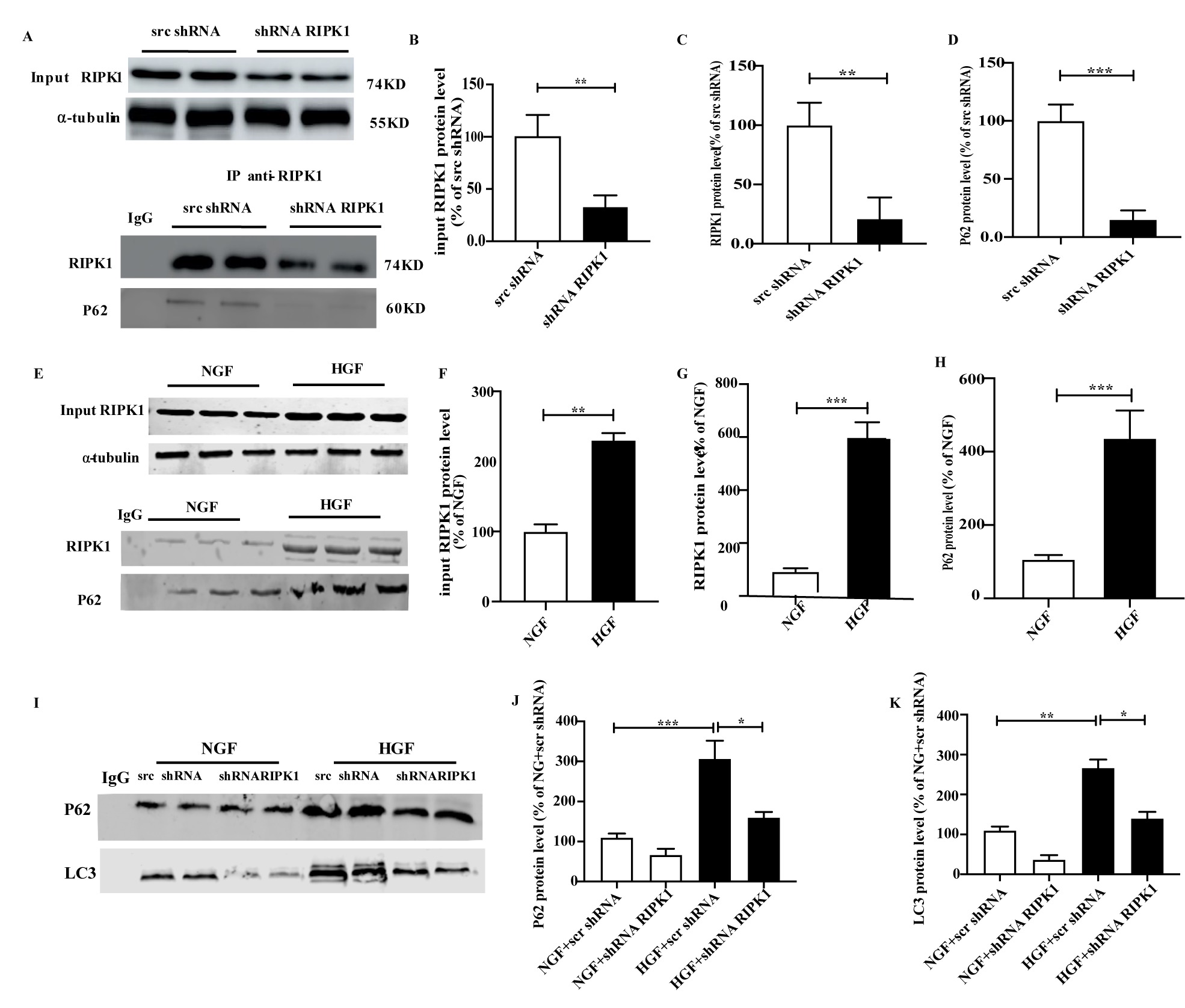

Supplement: Supplementary file 4 — Supplementary figure 4 [file 41419_2022_4587_MOESM4_ESM.jpg]

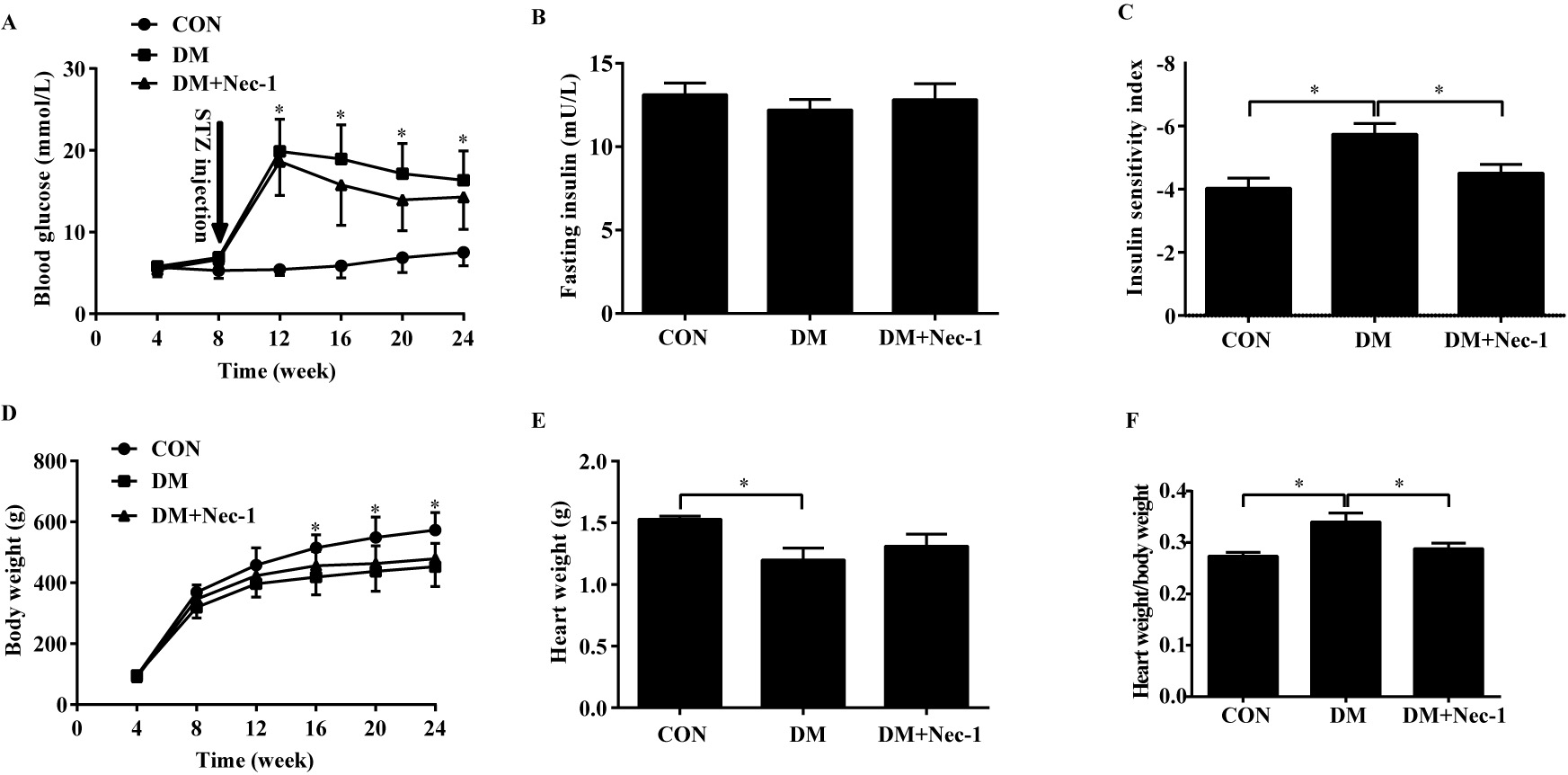

Supplement: Supplementary file 5 — Supplementary figure 5 [file 41419_2022_4587_MOESM5_ESM.jpg]
