# Supplementary material for: RIPK1-RIPK3 mediates myocardial fibrosis in type 2 diabetes mellitus by impairing autophagic flux of cardiac fibroblasts
Source: Cell Death Dis. 2022 Feb 14;13(2):147. doi: 10.1038/s41419-022-04587-1 (PMC8844355; doi:10.1038/s41419-022-04587-1)
Supplement: Supplementary file 6 — Supplementary figure Legend [file 41419_2022_4587_MOESM6_ESM.docx]

**Supplementary Figure1.** RIPK1/RIPK3 silencing alleviates HGF-induced fibrosis of CFs. LDH leakage, collagen I and collagen III levels, and OD values (A-D). Representative images and percentages of α-SMA (E-F) and ATP content (G) in RIPK1-knockdown CFs. LDH leakage, collagen I and collagen III levels, and OD values (H-K). Representative images and percentages of α-SMA (L-M) and ATP content (N) in RIPK3-treated CFs. n = 3 per group. Means ± SD. **P* < 0.05, ***P* < 0.01, ****P* < 0.001. NGF, normal glucose and fat group; HGF, high glucose and high fat group; OD, optical density; α-SMA, α smooth muscle actin; ATP, adenosine triphosphate.

**Supplementary Figure2.** MLKL silencing alleviates HGF-induced fibrosis of CFs. LDH leakage, collagen I and collagen III levels, and ATP content (A-D). n = 3 per group. Means ± SD. **P* < 0.05, ***P* < 0.01. NGF, normal glucose and fat group; HGF, high glucose and high fat group; ATP, adenosine triphosphate.

**Supplementary Figure3.** Nec-1 (100μM) and GSK (10μM) alleviates HGF-induced fibrotic of CFs. LDH leakage, collagen I, collagen III, α-SMA and ATP content in CFs treated with Nec-1 (100μM), GSK (10μM) (A-F) or the combination (G-L). n = 3 per group. Means ± SD. **P* < 0.05, ***P* < 0.01, ****P* < 0.001. HGF, high glucose and high fat group; Nec-1, necrostatin-1; GSK, GSK872; CQ, chloroquine; α-SMA, α smooth muscle actin; ATP, adenosine triphosphate.

**Supplementary Figure4.** Both the association of RIPK1 with P62 and the binding of P62 to LC3 were increased in HGF treated CFs, silencing of RIPK1 significantly decreased the association and binding. Representative images and percentages of IP of RIPK1 with P62 and the binding of P62 to LC3. n = 3 per group. Means ± SD. **P* < 0.05, ***P* < 0.01, ****P* < 0.001. NGF, normal glucose and fat group; HGF, high glucose and high fat group.

**Supplementary Figure5.** Nec-1 restores cardiac function in diabetic rats. Fasting blood glucose and insulin levels, body weights and heart weights (A-F). n = 10 per group. Mean ± SD, **P* < 0.05. CON, control group; DM, diabetic mellitus group.
